# Mn-CeO_2_ Nanomaterial for the Colorimetric Sensing of H_2_O_2_ and Ascorbic Acid

**DOI:** 10.3390/nano16070443

**Published:** 2026-04-07

**Authors:** Faxue Ma, Xiangju Wu, Zhen Ma, Jingjing Lu, Xueqing Zhu, Yuguang Lv

**Affiliations:** 1 College of Engineering, Huanghe S&T University, Zhengzhou 450046, China; 16692882324@163.com (X.W.); zzbb12388@163.com (Z.M.);; 2 National Engineering Research Center of Wheat and Corn Further Processing, School of Food Science and Technology, Henan University of Technology, Zhengzhou 450001, China; jjlu66@163.com

**Keywords:** Mn-CeO_2_, peroxidase activity, colorimetric sensing, H_2_O_2_, AA

## Abstract

Owing to the high stability and low cost of nanozymes, they have been extensively investigated and reported. In this work, highly active CeO_2_ nanoflowers were first prepared and then different metal elements were doped into the CeO_2_ nanoflower matrix via a novel synthesis method to fabricate M-CeO_2_ (M = Cu, Fe, Co, Mn, La) nanomaterials. Mn-CeO_2_ with the highest peroxidase-like activity was selected via systematic screening, the as-prepared Mn-CeO_2_ nanocomposites exhibited enhanced enzyme-like activity due to the strong metal-support interaction. This article explored the effects of doping ratio, pH, temperature, reaction time, and material concentration on its activity. A simple sensitive and selective colorimetric method was established and successfully used to detect hydrogen peroxide and ascorbic acid sensitively. When the hydrogen peroxide (H_2_O_2_) concentration is within the 2.0–120.0 μM range, the UV-visible absorbance at 652 nm was associated linearly with the H_2_O_2_ concentration, R^2^ = 0.9959, LOD = 1.7 μM (S/N = 3). The absorbance of the reaction system showed a good linear relationship with the ascorbic acid (AA) concentration (1.0–40.0 μM, R^2^ = 0.992), LOD = 0.98 μM (S/N = 3). This study provides an effective way to construct efficient nanozymes and their potential applications in sensing and detection.

## 1. Introduction

In recent decades, nanozyme preparation has been extensively investigated and reported owing to the high stability and low cost of nanozymes. Compared with natural enzymes, nanozymes possess superior advantages including stable activity, a large specific surface area, tunable catalytic activity, facile preparation, and low cost [1]. Among various nanozymes, CeO_2_ has garnered considerable attention due to its excellent oxygen storage capacity, mixed oxidation states of Ce^3+^ and Ce^4+^, and outstanding catalytic properties [2]. Therefore, designing nanocomposites based on CeO_2_ can provide a promising strategy to improve the overall catalytic performance of the material.

Morphological regulation and elemental doping are effective ways to regulate the activity of metal oxide nanozymes [3]. The Lu [4] research group has obtained the best adsorption capacity and photocatalytic activity by regulating different morphologies of CeO_2_ NRs, which can remove over 89.35% of tetracycline (TC) within 90 min; Fan [5] et al. studied the glucose sensing achieved by the application of Copper(II) oxide particles with three different forms (spheres, platelets, and spicules) in nonenzymatic glucose sensors. It was found that needle-shaped CuO nanoparticles exhibit the best morphology in this application. Wang et al. [6] also found that cobalt-doped Fe_3_O_4_ (Co@Fe_3_O_4_) nanozymes have more stronger peroxidase activity and a 100-fold higher affinity for H_2_O_2_ than Fe_3_O_4_ nanozymes. From an electronic structure perspective, the multiplicity of Mn’s oxidation states (Mn^2+^/Mn^3+^/Mn^4+^) can synergize with Ce’s Ce^3+^/Ce^4+^ redox pair to enhance electron transfer efficiency. This characteristic has been confirmed by multiple studies [7].

As important representatives of biological small molecules, H_2_O_2_ and AA play an important role in the living system. However, excessive H_2_O_2_ concentration produces toxic effects on cells, leading to cellular damage and tissue lesions [8]. AA is one of the common small biological molecules in human blood, with functions including enhancing immunity, increased capillary elasticity, and antioxidant and free radical scavenging. But when the human body lacks AA, it may cause a variety of diseases, such as rheumatoid arthritis, scurvy, joint pain, and so on [9]. Therefore, it is important to monitor the concentration changes of H_2_O_2_ and AA in biological systems.

Based on the above advantages, Mn doping can induce more oxygen vacancies in the CeO_2_ lattice, thus making Mn a suitable modifier for enhancing the catalytic performance of CeO_2_. The synergistic redox interplay between Ce^3+^/Ce^4+^ and Mn^2+^/Mn^3+^/Mnv is expected to form a self-sustaining electron transfer network that improves catalytic performance, as verified by XPS analysis in Section 3.1.3. Hydrogen peroxide and ascorbic acid play an irreplaceable role in the REDOX cycle in vivo. The human body generally obtains H_2_O_2_ and AA through food intake. This article adopts a new synthesis method to synthesize Mn-CeO_2_ for detecting H_2_O_2_ and AA in food, which can be effectively applied in food detection. We also comprehensively evaluated and explored the colorimetric biosensing properties of Mn-CeO_2_ nanocomposites. Compared to other sensors, Mn-CeO_2_ has a lower limit of detection (LOD) and a better linear range.

## 2. Materials and Methods

### 2.1. Materials

Cerium(III) nitrate hexahydrate (Ce(NO_3_)_3_·6H_2_O, AR), manganese (II) nitrate hexahydrate (Mn(NO_3_)_2_·6H_2_O, AR); sodium bicarbonate (NaHCO_3_, AR); 3,3′5,5′-tetramethylbenzidine (TMB, AR) and dimethylsulfoxide (DMSO, AR) were obtained from Aladdin Industrial Corporation (Shanghai, China). Hydrogen peroxide (H_2_O_2_, 30%), ethanol (C_2_H_5_OH, 95%), glacial acetic acid (CH_3_COOH, AR), and acetate (CH_3_COONa, AR) were bought from Sinopharm Chemical Reagent Co., Ltd. (Shanghai, China). Fructose (fru, 99.5%), glucose (Glu, AR), maltose monohydrate (mal, 97%), leucine (leu, 99%), glycocoll (gly, 98%), threonine (Thr, 98%), L-tryptophan (L-try, 95%), and cysteine (L-cys, 95%) were purchased from Sigma-Aldrich (Shanghai, China). All the chemicals were used as received without any further purification. Milli-Q water (18.2 mΩ·cm) was used throughout the experiments.

### 2.2. Synthesis of Mn-CeO_2_ Nano-Floral

Mn^2+^-doped CeO_2_ nanoflowers with different Mn^2+^ doping ratios (3%, 6%, 9%, 12%, 15%) were synthesized via a cold precipitation method. In each experiment, the molar ratio of Mn^2+^ to the total metal ions (Mn^2+^ + Ce^3+^) was calculated according to Equation (1) below. The obtained samples were labeled as 3% Mn-CeO_2_, 6% Mn-CeO_2_, 9% Mn-CeO_2_, 12% Mn-CeO_2_, and 15% Mn-CeO_2_, respectively.

(1) $$\mathrm{Mn}\;\%=\frac{\left[{\mathrm{Mn}}^{2+}\right]}{\left[{\mathrm{Mn}}^{2+}+{\mathrm{Ce}}^{3+}\right]}$$

For a typical synthesis of 3% Mn-CeO_2_ nanoflowers, 3.104 mM Ce (NO_3_)_3_·6H_2_O and 0.096 mM Mn(NO_3_)_2_·6H_2_O were dissolved in 200 mL distilled water and the mixture was stirred at 3 °C until complete dissolution. Subsequently, 0.78 g NaHCO_3_ was dissolved in 200 mL distilled water under stirring at 3 °C and this solution was rapidly poured into the above Ce(NO_3_)_3_·6H_2_O/Mn(NO_3_)_2_ mixed solution. Stirring was continued for 1 h and the mixture was allowed to stand at 3 °C for 24 h. The resulting precipitate was collected by centrifugation and washed three times with deionized water and absolute ethanol in sequence to remove residual ions. The washed precipitate was dried at 80 °C for 6 h and finally calcined in air at 450 °C for 4 h. The preparation method of pure CeO_2_ nano-floral is the same, with 3.2 mM Ce(NO_3_)_3_·6H_2_O as the precursor [10]. In order to compare the doping of different metals, representative different metal ions (M = Fe^3+^, Co^2+^, Cu^2+^, La^2+^) were selected and synthesized using the same method to obtain M-CeO_2_. The samples were labeled as 12 Fe-CeO_2_, 12 Co-CeO_2_, 12 Cu-CeO_2_, and 12 La-CeO_2_ according to the different doping metals.

### 2.3. Characterization

The chemical structure of the material was analyzed by X-ray diffraction (XRD, Rigaku Dmax-2000, Rigaku Corporation, Tokyo, Japan, Cu Kα radiation, 2θ 20° to 80°) at a scanning rate of 10°/min. Transmission electron microscopy (TEM) imaging was performed using a Hitachi HT7800 high-contrast transmission electron microscope (Hitachi, High-Tech Corporation, Tokyo, Japan). Scanning electron microscopy (SEM) observations were performed using a Hitachi Regulus-8100 field emission scanning electron microscope (Hitachi, High-Tech Corporation, Tokyo, Japan). Raman spectra were acquired using a confocal Raman spectrometer (Jobin-Yvon HR800, HORIBA Jobin Yvon, Paris, France). Brunauer-Emmett-Teller (BET) testing was performed on an AS-1C-TCD (Beijing Beifen-Ruili Analytical Instrument Co., Ltd., Beijing, China), while ultraviolet-visible absorption spectra were recorded using a TU-1901 spectrophotometer (Persee General Instrument Co., Ltd., Beijing, China).

### 2.4. Real Sample Pretreatment

Add 0.2 M NaAc-HAc buffer solution with pH = 4 to the commercial skimmed milk to cause sedimentation. After multiple centrifugations, take the supernatant, remove impurities, and dilute with ultrapure water to further avoid interference from impurities. Add different concentrations of H_2_O_2_ solution to the diluted skimmed milk to obtain labeled milk. Perform the detection of H_2_O_2_ content in the milk according to the determination steps for H_2_O_2_, using 2000 μL of 0.2 M NaAc-HAc buffer solution (pH = 4), 100 μL of 12Mn-CeO_2_ (0.4 mg mL^−1^), 100 μL of TMB (10 mM), and 100 μL of labeled milk of different concentrations (50, 100, 150 μM). Shake well and react at 50 °C for 20 min. Perform three parallel determinations of the absorbance at 652 nm for each system and record the values. For the analysis of commercial vitamin C tablets (labeled content: 100 mg per tablet), sample pretreatment was performed following the procedures specified in the Chinese Pharmacopoeia (2020 Edition, Part II). Briefly, the tablets were homogenized and dissolved in ultrapure water to prepare a 100 mM stock solution, which was further diluted to 1 mM immediately before the analysis. The spiking levels of AA in the tablet matrix were set at 10, 20, and 30 μM, respectively Throughout all assays, the concentrations of TMB and Mn-doped CeO_2_ nanoparticles were maintained consistently with those used in the milk analysis. All measurements were performed in triplicate to ensure analytical reproducibility.

## 3. Results

### 3.1. Characterization of the M-CeO_2_ Material

#### 3.1.1. XRD Characterization Analysis

**Figure 1:**
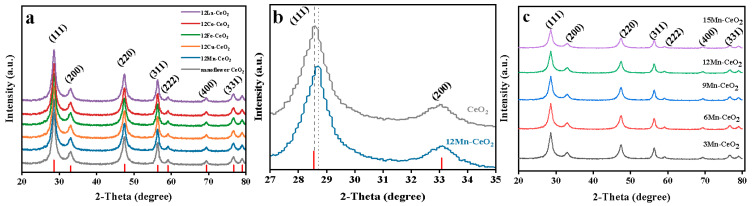
(**a**) XRD patterns of pure CeO_2_ and 12% M-CeO_2_ (M = Cu, Fe, Co, Mn, La); (**b**) magnified XRD patterns of CeO_2_ and 12% Mn-CeO_2_ (111) crystal plane (the meaning of the dotted line is the standard diffraction peak position of the CeO_2_ (111) crystal plane). (**c**) XRD patterns of Mn-CeO_2_ nanocomposites with different doping ratios.

Figure 1a presents the XRD patterns of pure CeO_2_ and 12% M-CeO_2_ (M = La, Fe, Co, Cu, Mn) samples. All diffraction peaks (2θ = 28.6°, 33.1°, 47.5°, 56.3°, 59.1°, 69.4°, 76.7°, 79.1°) were in good agreement with the standard cubic fluorite structure of CeO_2_ (JCPDS No. 34-0394) and no characteristic peaks of impurity metal oxides (e.g., Mn_3_O_4_, Fe_2_O_3_, CuO) are observed. This result confirms that all transition metal ions are successfully doped into the CeO_2_ lattice to form a single-phase solid solution, without the formation of separate metal oxide phases.

To further investigate the lattice change of Mn-doped CeO_2_, the (111) crystal plane (the strongest diffraction peak of CeO_2_) was magnified for analysis (Figure 1b). The (111) peak of 12% Mn-CeO_2_ shifts to a higher 2θ value (28.7°) compared with pure CeO_2_ (28.6°), and the diffraction peak shows slight broadening [11]. The peak shift and broadening imply the occurrence of lattice contraction and distortion in CeO_2_ after Mn doping and the specific structural change was quantitatively characterized by lattice parameter calculation (see Table 1).

Figure 1c shows the XRD patterns of Mn-CeO_2_ nanocomposites with different Mn doping ratios (3%, 6%, 9%, 12%, 15%). All diffraction peaks are consistent with the cubic fluorite structure of CeO_2_ (JCPDS No. 34-0394), confirming the successful formation of Mn-CeO_2_ single-phase solid solutions at all doping ratios. With the increase in Mn doping content, the diffraction peaks gradually shift to higher 2θ values, broaden continuously, and the peak intensity decreases progressively.

Based on Bragg’s law (2dsinθ = nλ) and the cubic crystal system lattice parameter formula $d=\frac{a}{\sqrt{h^{2}+k^{2}+l^{2}}}$ (a = lattice parameter, hkl = crystal plane index), the lattice parameters of pure CeO_2_ and Mn-CeO_2_ with different doping ratios were calculated using the (111) crystal plane data and the results are summarized in Table 1. The lattice parameter of pure CeO_2_ is 5.411 Å, while that of 12% Mn-CeO_2_ decreases to 5.402 Å; with the increase in Mn doping ratio (3–15%), the lattice parameter of Mn-CeO_2_ decreases gradually (3%: 5.409 Å, 6%: 5.407 Å, 9%: 5.404 Å, 15%: 5.399 Å). This quantitative result directly verifies that the small-radius Mn^2+^ (0.83 Å) replaces Ce^4+^ (1.01 Å) in the CeO_2_ lattice, leading to significant local lattice contraction.

The gradual broadening of diffraction peaks is attributed to the reduced crystallite size and increased lattice strain caused by Mn doping, while the decrease in peak intensity is due to the decreased crystallinity of CeO_2_ and the difference in X-ray scattering factors between Mn and Ce elements. No characteristic peaks of manganese oxides (e.g., Mn_3_O_4_, MnO_2_) are detected in all samples, which further confirms the formation of homogeneous Mn-CeO_2_ solid solutions instead of phase-separated composites. The lattice contraction and distortion induced by Mn doping effectively increase the exposure of active sites and promote charge transfer in the material, which is the structural basis for the enhanced peroxidase-like activity of Mn-CeO_2_.

#### 3.1.2. SEM Characterization Analysis

**Figure 2:**
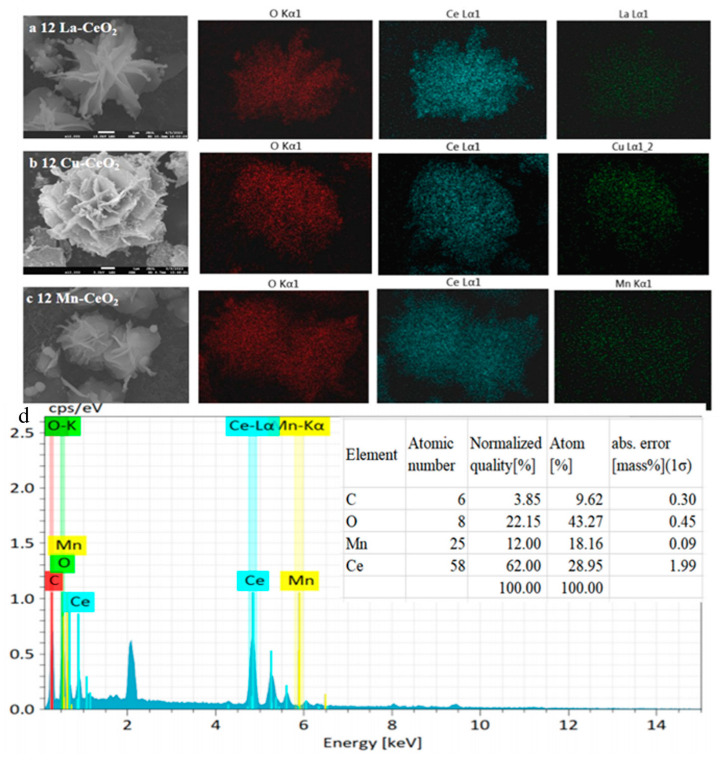
The SEM and mapping plots of M-CeO_2_ (**a**) 12 La-CeO_2_, (**b**) 12 Cu-CeO_2_, (**c**) 12 Mn-CeO_2_, and (**d**) EDS spectrum and elemental composition analysis of 12 Mn-CeO_2_.

Scanning electron microscopy (SEM) was used to observe the microstructure and distribution of synthetic materials and energy dispersive spectroscopy (EDS) was used to study the distribution of elements. The element mapping results under the mapping mode of SEM are shown in Figure 2. Doping with transition metal elements did not alter the morphology of CeO_2_, which still retained a well-defined nanoflower structure. The transition metal ions were found to be uniformly distributed on the CeO_2_ nanoflowers. The relative signal intensities of the Ce and transition metal element mappings confirmed the successful preparation of La-CeO_2_, Cu-CeO_2_, and Mn-CeO_2_ nanocomposites. The thickness of the flower petals of 12% La-CeO_2_ slightly increased, while the looseness of the petals showed no significant change. The overall morphology was closest to that of pure CeO_2_. The flower petals of 12% Cu-CeO_2_ became thinner and slight pore structures appeared between the petals. The flower petals of 12% Mn-CeO_2_ were the thinnest, with finer branches and a significantly increased looseness between the petals, forming more mesoporous structures. This microscopic morphological feature exposed more active sites, which is also an important structural reason for its optimal catalytic activity. These results provide fundamental structural and elemental distribution support for the subsequent screening and evaluation of CeO_2_ with optimal metal doping for enhanced peroxidase-like activity. Energy dispersive spectroscopy (EDS) was employed to analyze the elemental composition of 12% Mn-CeO_2_ nanocomposites. The EDS spectrum (Figure 2d) clearly shows the characteristic peaks of Ce, O, and Mn, confirming the successful incorporation of Mn into the CeO_2_ matrix. It should be noted that quantitative analysis of light elements (e.g., C and O) by EDS is subject to significant uncertainties due to low-energy X-ray absorption effects and potential surface contamination [12]. Therefore, the Mn/Ce atomic ratio was used as the primary indicator for quantifying the doping level, as both Mn and Ce are heavier elements with higher EDS quantification accuracy [13]. The atomic ratio of Mn/(Mn + Ce) is calculated to be approximately 38.5%, confirming that Mn is effectively doped into the CeO_2_ lattice. This value is consistent with the nominal doping level (12%) when considering the relative sensitivity factors.

#### 3.1.3. XPS Characterization Analysis

**Figure 3:**
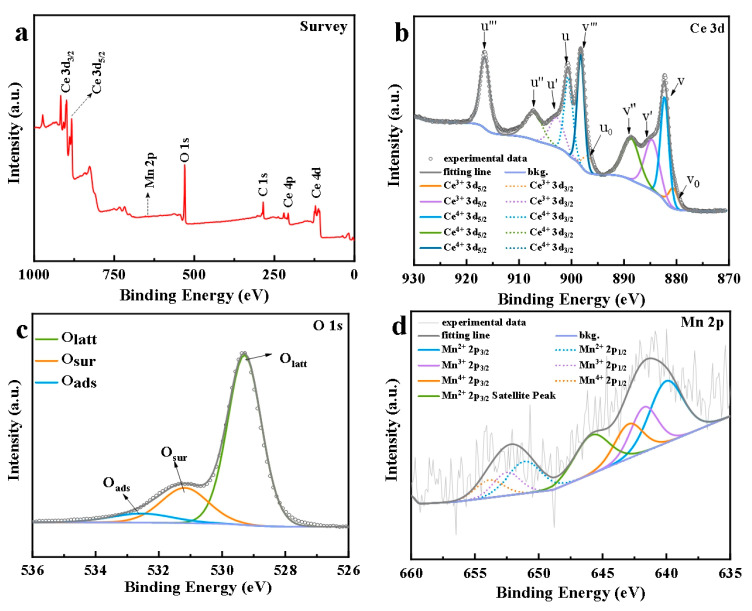
The XPS spectra of Mn-CeO_2_ (**a**) survey, (**b**) Ce 3d, (**c**) O1s, and (**d**) Mn 2p.

The surface elemental composition and chemical state of Mn-CeO_2_ nanocomposites were analyzed by X-ray photoelectron spectroscopy (XPS). Figure 3a shows the full XPS spectrum of the sample. From Figure 3a, we can see that there are four elements on the surface of the material: Ce, Mn, O, and C. C comes from adsorbed CO_2_ or other carbon-containing organic compounds in the air, further proving that the synthesized composite material contains Mn and CeO_2_. Figure 3b shows the high-resolution XPS spectra of Ce 3d with multiple peaks. Due to the hybridization between the 4f energy level of Ce and the 2p valence band of O, the Ce 3d are relatively complex, with five pairs of spin-coupled orbitals. These peaks are decomposed into 10 sub-peaks with good resolution through curve fitting. Ce 3d_3/2_ and Ce 3d_5/2_ are shown using *u* and *v*, respectively, where *u′*, *v′*, *u*_0_, and *v*_0_ correspond to Ce^3+^, *u″*, *v″*, *u′, v′*, and *u′*. The six peaks of v correspond to Ce^4+^, indicating that both Ce^3+^ and Ce^4+^ exist in the crystal of the composite material and Ce^4+^ plays a dominant role. With the addition of Mn, the content of Ce^3+^ can be calculated using a semi-quantitative formula [11], as follows in Equation (2).

(2) $$Ce^{3+}=\frac{\left(v_{0}+v^{\prime }+u_{0}+u^{\prime }\right)}{\left(v_{0}+v+v^{\prime }+v^{\prime \prime }+v^{\prime \prime \prime }+u_{0}+u+u^{\prime }+u^{\prime \prime }+u^{\prime \prime \prime }\right)}$$

From this, it was calculated that the Ce^3+^ content in CeO_2_ decreased by 27.9%. In Pirmohamed’s [14] study, they proposed that the lower the Ce^3+^ content in CeO_2_, the stronger the catalase-like ability of the material. So, in our experiment, reducing the amount of Ce^3+^ can help the catalyst convert H_2_O_2_ into ·OH, thereby promoting the enhancement of the material’s peroxidase activity. Figure 3c shows the O1s spectrum in Mn-CeO_2_. After peak fitting, it is confirmed that the crystal contains three types of oxygen: lattice oxygen (Olatt), surface oxygen (Osur), and adsorbed oxygen (Oads). This indicates that the nanocomposite material has excellent oxygen storage capacity and good catalytic oxidation activity. At the same time, there are two prominent peaks in the Mn 2p orbital spectra shown in Figure 3d, with binding energies of 641.3 and 654.2 eV, respectively, corresponding to the Mn 2p_3/2_ and 2p_1/2_ orbitals. The peak fitting treatment shows that Mn^2+^, Mn^3+^, and Mn^4+^ coexist in the nanocomposites and the dominant content of Mn^2+^ is close to 48.8%. Mn^3+^ and Mn^4+^ account for 51.2%, indicating a robust interfacial interaction between Mn and CeO_2_ nano-floral.

#### 3.1.4. Raman Test Analysis

**Figure 4:**
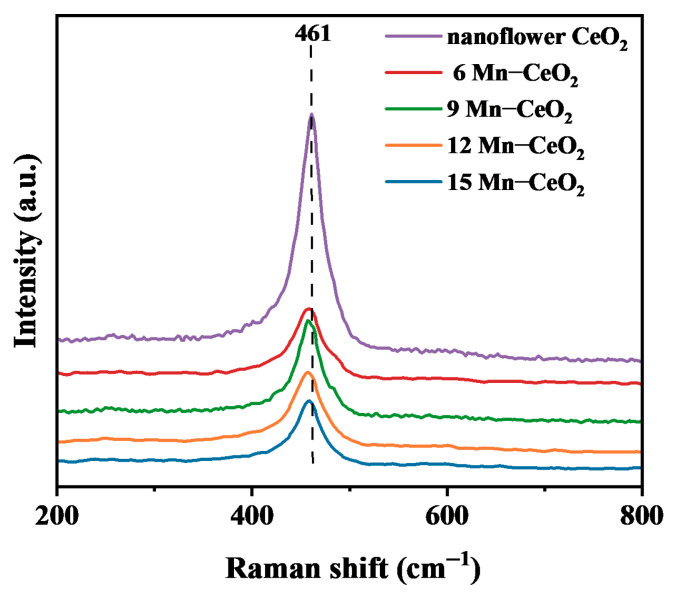
Raman spectra of CeO_2_ and Mn-CeO_2_.

The introduction of dopants causes a shift in the position of the lattice Raman vibration peak. Figure 4 shows the Raman spectra of flower-like CeO_2_ and Mn-CeO_2_ samples with different doping ratios. For the pure CeO_2_ sample, the F_2g_ Raman-active mode of the cubic fluorite structure exhibits a strong peak at 461 cm^−1^, which is attributed to the symmetric breathing mode of the oxygen atoms surrounding the cerium ions. With the incorporation of Mn^2+^, compared to pure CeO_2_, the peak intensity of the Mn-CeO_2_ samples is significantly reduced, the peak broadens, and a red shift occurs to 457–458 cm^−1^. Generally, a decrease in grain size and an increase in specific surface area affect the position of the Raman F_2g_ peak [15]. Therefore, the results from XRD, BET, and Raman clearly demonstrate that the smaller grain size and increased specific surface area are likely the causes of the F_2g_ red shift. The red shift can be attributed to Mn^2+^ replacing the main lattice in CeO_2_, leading to electron-vibrational coupling. These changes should be attributed to Mn^2+^ doping; another reason for the peak broadening may be the increase in oxygen vacancies, which is related to structural defects arising from the partial incorporation of Mn into the CeO_2_ lattice. In Mn-CeO_2_ samples, additional oxygen vacancies are generated to compensate for the valence mismatch between Mn^2+^ and Ce^4+^ ions [16].

#### 3.1.5. Adsorption Isothermal Curve Testing

**Figure 5:**
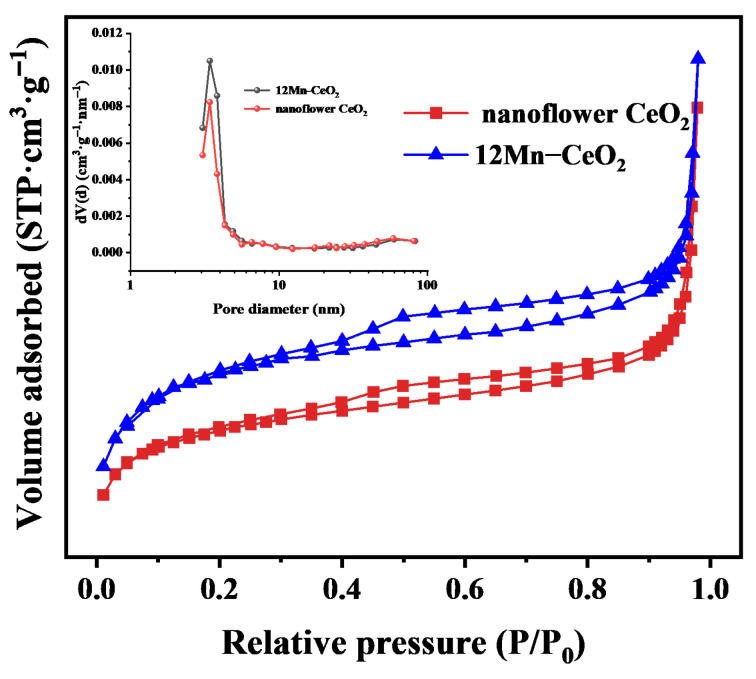
N_2_ adsorption and desorption curves and pore size distributions for CeO_2_ and 12Mn-CeO_2_.

Figure 5 shows the nitrogen adsorption-desorption isotherms and pore size distribution profiles of the prepared flower-like CeO_2_ and 12Mn-CeO_2_. According to the IUPAC and Brunauer-Deming-Teller classifications, the isotherms of the prepared samples are classified as Type IV isotherms with H_2_-type hysteresis, indicating the presence of a relatively uniform mesoporous structure in the samples. Table 2 lists the specific surface area (SBET), pore volume (VP), and maximum pore diameter (dp) derived from the nitrogen adsorption-desorption isotherms. Compared to the flower-like CeO_2_ sample, the specific surface area of the prepared 12Mn-CeO_2_ sample has increased. This phenomenon may be related to the dispersion of the oxide promoted by Mn doping into the fluorite structure of CeO_2_. Consequently, more active sites were created, effectively enhancing peroxidase-like activity. The maximum pore diameters of the flower-like CeO_2_ and 12Mn-CeO_2_ samples were 3.412 nm and 3.411 nm, respectively, further confirming the presence of a mesoporous structure in the samples.

### 3.2. Peroxidase-like Activity Evaluation of M-CeO_2_ Composites

#### 3.2.1. Peroxidase-like Activity

**Figure 6:**
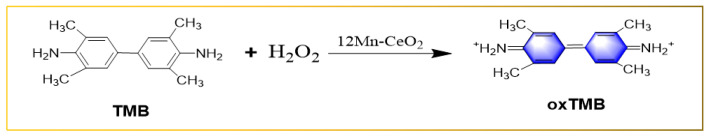
Mechanism of oxidation of TMB catalyzed by 12 Mn-CeO_2_.

**Figure 7:**
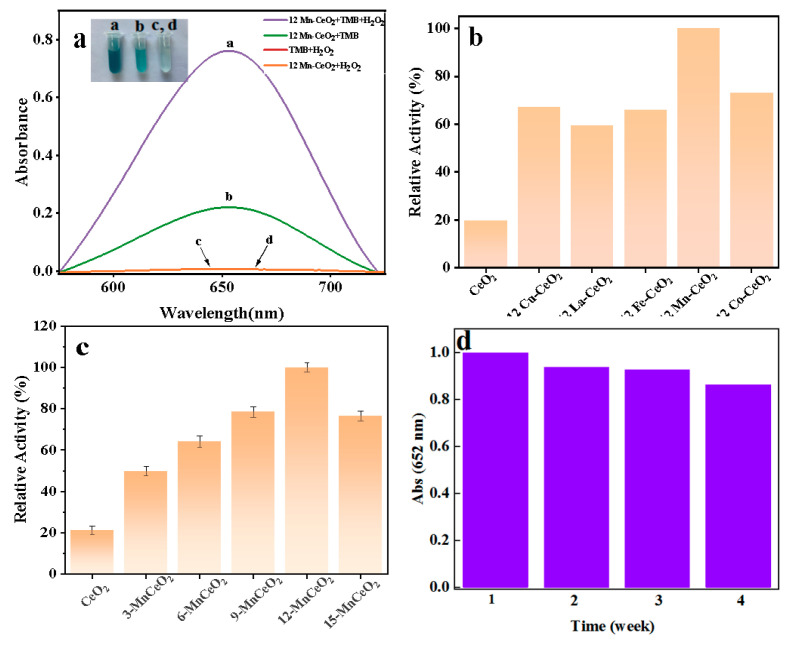
(**a**) UV-Vis absorption spectra of the different reaction systems. (**b**) Comparison of the catalytic activities of the different 12% M-CeO_2_ catalysts. (**c**) Comparison of the catalytic activities of Mn-CeO_2_ doped catalysts with different proportions. (**d**) Stability assessment of 12% Mn-CeO_2_.

To verify the peroxidase-like activity of synthesized nanozymes, a standardized catalytic system was established using TMB as the chromogenic substrate in NaAc-HAc buffer (pH 4.0). The catalytic mechanism follows the Fenton-like principle (Figure 6): M-CeO_2_ catalyzes H_2_O_2_ decomposition to generate ·OH radicals, which subsequently oxidize colorless TMB to blue oxTMB (λₘₐₓ = 652 nm). As shown in Figure 7a, the complete reaction system (12% Mn-CeO_2_-TMB-H_2_O_2_) exhibited strong absorption at 652 nm, whereas control systems lacking any single component (H_2_O_2_, catalyst, or TMB) showed negligible absorption (<5% of complete system). This strict H_2_O_2_-dependent behavior confirms the intrinsic peroxidase-mimicking nature of Mn-CeO_2_, analogous to natural horseradish peroxidase (HRP) [17].

On this basis, to screen the metal element with the best doping effect, 12% of different transition metal ions (M = Mn^2+^, Cu^2+^, Fe^3+^, Co^2+^, La^2+^) were doped into nanoflower-like CeO_2_ and the peroxidase-like activities of the prepared M-CeO_2_ composites were compared under the same reaction conditions above, as shown in Figure 7b. The doping of all transition metals could improve the catalytic activity of pure CeO_2_ and the catalytic activity showed a trend of Mn-CeO_2_ > Co-CeO_2_ > Cu-CeO_2_ > Fe-CeO_2_ > La-CeO_2_. Among them, Mn-CeO_2_ had the highest catalytic activity, which was five times that of pure CeO_2_, confirming the unique synergistic catalytic effect between Mn ions and CeO_2_ nanoflowers.

Furthermore, the effects of different Mn^2+^ doping levels (3%, 6%, 9%, 12%, 15%) on the peroxidase-like activity of Mn-CeO_2_ composites were investigated to determine the optimal doping ratio, as shown in Figure 7c. The material exhibited the highest catalytic activity at the Mn^2+^ doping level of 12%, which was attributed to the highly synergistic interaction between the electron-hole pairs provided by Mn^2+^ and the Ce^3+^/Ce^4+^ redox pair in CeO_2_ at this ratio, thus significantly enhancing the electron transfer rate during the catalytic process. Therefore, 12% Mn-CeO_2_ was selected as the peroxidase-mimetic nanozyme for all subsequent experiments.

In addition, the storage stability of 12% Mn-CeO_2_ was also investigated to evaluate its practical application potential, as shown in Figure 7d. The catalytic activity of the material remained above 86% after continuous testing for 4 weeks, indicating that 12% Mn-CeO_2_ exhibited excellent storage stability and could maintain high catalytic activity over a long period of time.

#### 3.2.2. Optimization of Activity Conditions

**Figure 8:**
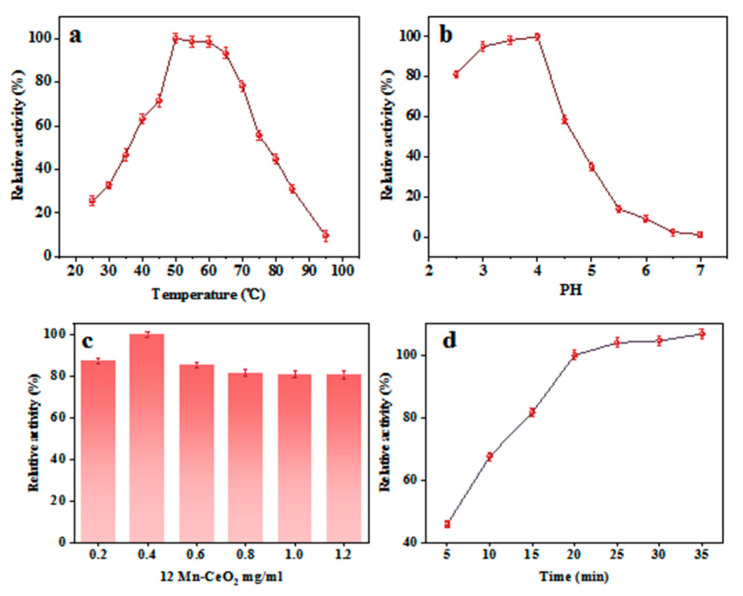
Effect of different conditions on the Mn-CeO_2_ peroxidase activity: (**a**) temperature, (**b**) pH, (**c**) amount of catalyst, and (**d**) reaction time.

In order to maximize the catalytic capacity of Mn-CeO_2_, the catalytic conditions pH, temperature, time, and catalyst dosage were studied and optimized. Set the absorbance value of the maximum enzyme-like activity to 100%, record the ratio of other enzyme activities to it as relative activity, calculate the average of three parallel measurements for each experiment as the final result, and calculate its relative standard deviation. As shown in Figure 8a, when the temperature is controlled within the range of 25–95 °C, the catalytic activity of Mn-CeO_2_ shows a trend of increasing and then decreasing, reaching its maximum activity at 50 °C. When the temperature is above a certain value, the catalytic activity decreases, which is caused by the instability of TMB at high temperatures. Notably, Mn-CeO_2_ maintains over 60% relative activity across a broad temperature range of 40–70 °C. This stands in stark contrast to natural enzymes, which typically function only within a narrow, mild temperature window, highlighting the nanocatalyst’s superior adaptability to practical environmental conditions.

Figure 8b explores the effect of pH on the catalytic activity of Mn-CeO_2_ and finds that the catalytic activity under weak acid conditions is higher than that under neutral and alkaline conditions, consistent with most nanoenzyme reports. This is because the acidic reaction system is conducive to the formation of oxTMB. The maximum catalytic activity was achieved at a pH of 4.0, so subsequent experiments were conducted at a pH of 4.0.

At the same time, to investigate the effect of catalyst dosage on catalytic activity, the absorbance of catalyst dosage ranging from 0.2 to 1.2 mg·mL^−1^ was tested, as shown in Figure 8c. The maximum catalytic activity could be achieved when the catalyst dosage was 0.4 mg·mL^−1^. Therefore, the optimal catalyst dosage was determined to be 0.4 mg·mL^−1^.

Under optimal conditions at pH = 4.0, we investigated the effect of reaction time on the system’s absorbance. As shown in Figure 8d, the absorbance of the Mn-CeO_2_-TMB-H_2_O_2_ system increased rapidly over 5 to 20 min, then leveled off after 20 min. Considering detection efficiency, 20 min was selected as the reaction time for subsequent detection experiments. In summary, we have determined the optimal reaction conditions for subsequent experiments as follows: temperature 50 °C, pH = 4.0, Mn-CeO_2_ catalyst dosage 0.4 mg·m^−1^, and reaction time 20 min.

#### 3.2.3. Kinetic Studies and the Mechanism of Peroxidase-like Activity

**Figure 9:**
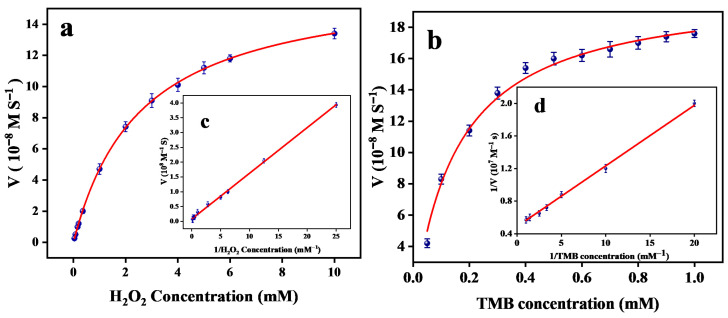
Experimental curves of the steady-state kinetics of the Mn-CeO_2_ nanozymes: (**a**) kinetic curve of 0.5 mM TMB and the double-reciprocal curve (**c**); 8 mM H_2_O_2_ kinetic curve (**b**) and double reciprocal curve (**d**).

The catalytic kinetic process of Mn-CeO_2_ was studied using the principle of enzyme catalysis kinetics under the optimal reaction conditions mentioned above. In NaAc-HAc (pH = 4.0, 0.2 M) buffer solution, fix Mn-CeO_2_ at a termination concentration of 20 μg·mL^−1^, TMB concentration of 0.5 mM, and change H_2_O_2_ concentration (1~10 mM). Similarly, in NaAc-HAc (pH = 4.0, 0.2 M) buffer solution, fix the termination concentration of Mn-CeO_2_ to 20 μg·mL^−1^, with an H_2_O_2_ concentration of 8 mM, the kinetic curves of the two substrates H_2_O_2_ and TMB were detected by changing the TMB concentration (0.05~1 mM). From Figure 9a,c, it can be seen that Mn-CeO_2_ conforms to the classic Michaelis-Menten curve. By fitting the Lineweaver-Burk double reciprocal plots (Figure 9b,d), the corresponding Michaelis constant (K_m_) and maximum reaction rate (V_max_) were calculated, with results summarized in Table 3. The K_m_ value reflects the affinity between the enzyme and substrate; a lower K_m_ indicates stronger affinity. Table 1 shows that when TMB serves as the substrate, Mn-CeO_2_ exhibits a K_m_ value of 0.156 mM, lower than the 0.434 mM value for native horseradish peroxidase (HRP), indicating Mn-CeO_2_ possesses higher affinity for the TMB substrate. When using H_2_O_2_ as the substrate, Mn-CeO_2_ exhibits a K_m_ value of 2.588 mM, indicating that a higher H_2_O_2_ concentration is required to achieve the maximum reaction rate. This may be related to its catalytic sites and reaction pathways. Furthermore, Mn-CeO_2_ exhibited higher V_max_ values for both substrates compared to reference nanocatalyst materials (e.g., Fe_3_O_4_ MNPs and 6Fe/CeO_2_), further validating its superior catalytic efficiency.

Comparison with the literature nanozymes: the kinetic parameters of Mn-CeO_2_ were systematically compared with representative nanozymes reported in the literature (Table 1). Notably, Mn-CeO_2_ exhibited a significantly lower Kₘ value for H_2_O_2_ (2.588 mM) compared to Fe_3_O_4_ MNPs (154 mM) [18] and 6Fe/CeO_2_ (47.6 mM) [19], indicating enhanced substrate affinity due to Mn doping. This superior affinity is attributed to the multi-valence nature of Mn (Mn^2+^/Mn^3+^/Mn^4+^), which forms a more flexible redox cycle with Ce^3+^/Ce^4+^ than the limited Fe^2+^/Fe^3+^ cycling in Fe_3_O_4_ [18]. Furthermore, the Vₘₐₓ values of Mn-CeO_2_ (16.89 × 10^−8^ M·s^−1^ for H_2_O_2_, 20.50 × 10^−8^ M·s^−1^ for TMB) are higher than those of 6Fe/CeO_2_ (8.6 and 16.6 × 10^−8^ M·s^−1^) [19], demonstrating superior catalytic efficiency. The enhanced performance of Mn-CeO_2_ over Fe-doped counterparts highlights the advantage of Mn doping in facilitating electron transfer [6].

#### 3.2.4. The Catalytic Mechanism of Mn-CeO_2_

**Figure 10:**
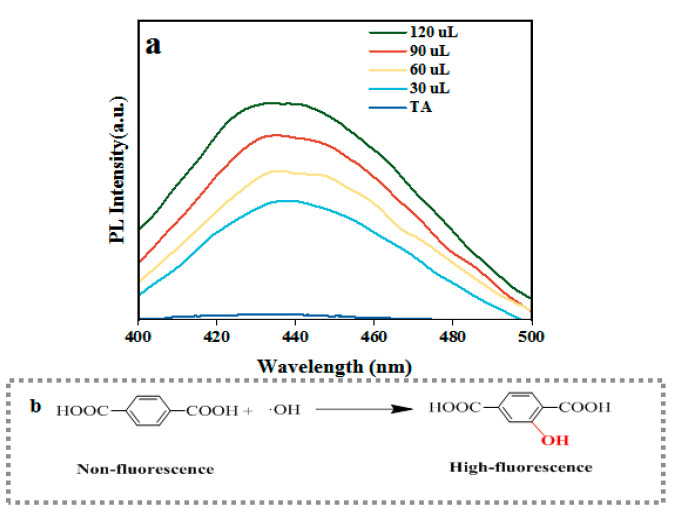
(**a**) Exploring the catalytic oxidation mechanism of 12 Mn-CeO_2_ with TA as a fluorescent probe, (**b**) ·OH induced the conversion of non-fluorescent terephthalic acid to highly fluorescent 2-hydroxyterephthalic acid.

12 Mn-CeO_2_ may follow the Fenton-like principle when simulating peroxidase activity, that is, under acidic conditions, peroxidase-like enzymes can catalyze the decomposition of H_2_O_2_ to generate ·OH, which in turn oxidizes colorless TMB to produce blue oxTMB. This study used non-fluorescent terephthalic acid (TA) for experiments to investigate whether the detection mechanism is related to the generation of ·OH, that is, when TA combines with ·OH, it will generate highly fluorescent 2-hydroxyterephthalic acid (2OH-TA). From Figure 10a, it can be seen that at an excitation wavelength of 320 nm, the 12 Mn-CeO_2_-TA-H_2_O_2_ system produces fluorescence at 435 nm, and the fluorescence intensity of the reaction system also increases with the increase in 12 Mn-CeO_2_ content. This phenomenon indicates that the mechanism of action of 12 Mn-CeO_2_ as a peroxidase mimetic enzyme can be attributed to the production of ·OH. Figure 10b explains the catalytic mechanism.

**Figure 11:**
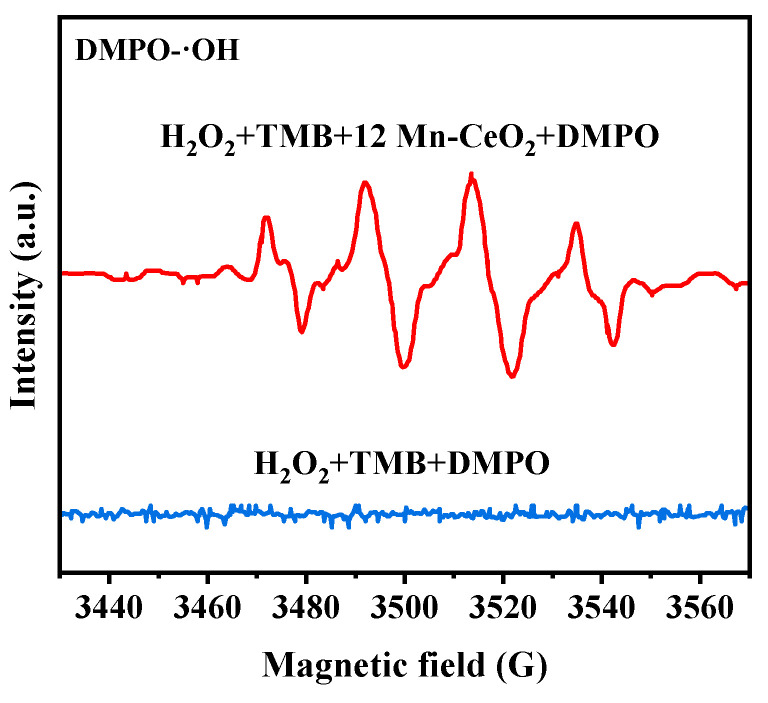
The ESR spectra of the different reaction systems.

In order to further prove that the action mechanism of 12 Mn-CeO_2_ as a peroxidase mimetic enzyme comes from the production of ·OH, because the ESR method is considered a reliable method to characterize free radicals, it is proved by the ESR method that 12 Mn-CeO_2_ can catalyze H_2_O_2_ to produce OH. Spin trap DMPO can capture OH and form a typical DMPO/·OH spin adduct with an ESR signal peak of 1:2:2:1 relative intensity. Figure 11 shows the characteristic ESR signal of DMPO/·OH in the presence of 12 Mn-CeO_2_ and H_2_O_2_; however, in the absence of 12 Mn-CeO_2_, there is no ESR signal in the reaction system. This result provides strong evidence that the mechanism by which Mn-CeO_2_ mimics peroxidase indeed stems from its ability to catalyze the generation of ·OH radicals from H_2_O_2_.

### 3.3. H_2_O_2_ Detection Using 12 Mn-CeO_2_

**Figure 12:**
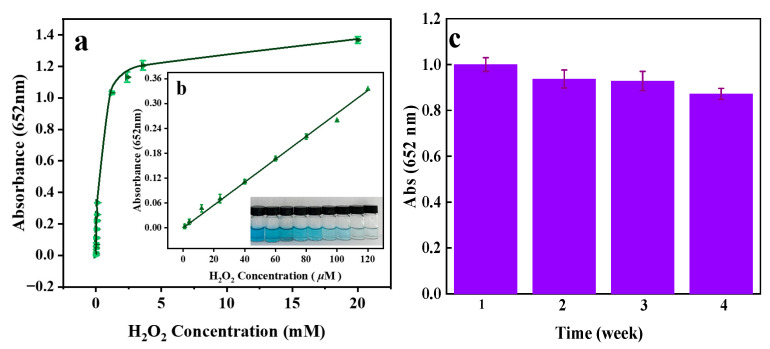
(**a**) Concentration change curve of the test H_2_O_2_. (**b**) Standard curve for detecting H_2_O_2_. (**c**) Repeatability and stability of the H_2_O_2_ were examined.

As an important representative of small biological molecules, H_2_O_2_ plays an important role in life systems. A colorimetric detection method for H_2_O_2_ was established by selecting Mn-CeO_2_ in this experiment, as shown in Figure 12a. In the H_2_O_2_ concentration range of 1 μM~20 mM, the corresponding absorbance increases with the increase in H_2_O_2_ concentration. As shown in Figure 12b, when the H_2_O_2_ concentration is within the 2.0~120.0 μM range, the UV-visible absorbance at a wavelength of 652 nm is linearly correlated with the concentration of H_2_O_2_. The linear equation is (R^2^ = 0.9959): A = 0.0027C_H2O2_ + 0.0059. The detection limit of H_2_O_2_ is determined using the formula LOD = 3.3 δ/S and it is found to be 1.7 μM. The ultra-low LOD of Mn-CeO_2_ for H_2_O_2_ detection (1.7 μM) compared with other nanozymes in Table 4 is largely attributed to the unique nanoflower-like morphology of the prepared material, which shows distinct structural advantages over the spherical (e.g., CuO spheres) and rod-like (e.g., CeO_2_ NRs) morphologies mentioned in the introduction. First, the nanoflower-like Mn-CeO_2_ has a three-dimensional hierarchical structure with ultra-thin petals, fine branches, and significantly increased inter-petal looseness (confirmed by SEM characterization in Section 3.1.2), which forms abundant mesoporous structures and a larger specific surface area compared with the compact spherical or rod-like morphologies. This structural feature maximizes the exposure of active sites introduced by Mn-doped solid solution (including Ce^3+^/Ce^4+^ and Mn^2+^/Mn^3+^/Mn^4+^ redox pairs), while spherical and rod-like metal oxides usually have limited exposed active sites due to their smooth or dense surface characteristics. Second, the mesoporous structure of nanoflowers greatly accelerates the mass transfer rate of reaction substrates (H_2_O_2_ and TMB) to the active sites of the material, which shortens the reaction time of ·OH generation and TMB oxidation, and thus improves the response sensitivity of the colorimetric system to low concentrations of H_2_O_2_. In contrast, the relatively closed structure of spherical/rod-like morphologies hinders the contact between substrates and internal active sites, leading to lower catalytic efficiency and higher LOD. Third, the nanoflower-like hierarchical structure effectively avoids the agglomeration of nanomaterials, which maintains the high dispersibility of Mn-CeO_2_ in the reaction system and ensures the full play of catalytic activity, while spherical nanoparticles are prone to agglomeration, and rod-like materials have poor dispersibility in solution, both of which will reduce the actual catalytic efficiency of the material. In summary, the nanoflower-like morphology of Mn-CeO_2_ constructs a structural foundation for efficient catalytic reaction by increasing active site exposure, accelerating mass transfer and maintaining good dispersibility, which is the key structural reason for the material to achieve a lower LOD than most spherical/rod-like nanozyme sensors for H_2_O_2_ detection.

Simultaneously, the reproducibility and stability of the material were evaluated. As shown in Figure 12c, measurements were taken weekly, and cyclic performance tests were conducted. Continuous monitoring over 4 weeks revealed that the material’s activity remained above 87.2%, indicating excellent reproducibility and stability. The comparison of H_2_O_2_ and LOD ranges detected by nano-enzymes of different materials is shown in Table 5. To validate the feasibility of this method in practical sample analysis, we applied it to the detection of H_2_O_2_ in skim milk and conducted spiked recovery experiments to assess the method’s accuracy and precision. The results are shown in Table 3. The recoveries at three different spiking levels ranged from 98.4% to 102.8%, with relative standard deviations (RSDs, *n* = 3) of 1.78% to 2.25%. These results demonstrate that the Mn-CeO_2_-based colorimetric sensing method is accurate and reliable for the determination of H_2_O_2_ in real samples.

### 3.4. AA Detection Using Mn-CeO_2_

**Figure 13:**
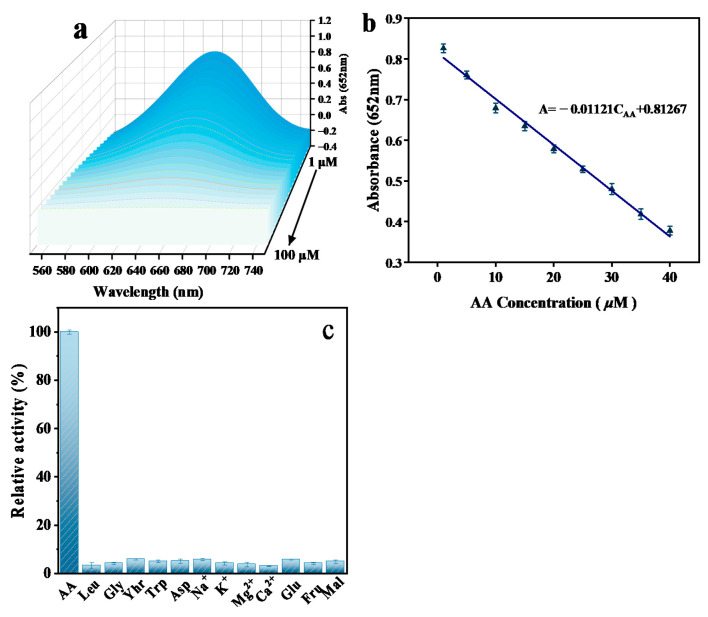
(**a**) UV-visible absorption spectrum changes with AA concentration; (**b**) concentration response curve for detecting AA and corresponding linear calibration map; (**c**) selectivity study of 12% Mn-CeO_2_ for AA detection in the presence of interfering substances (Leu, Gly, Thr, L-Cys, Trp, Na^+^, K^+^, Mg^2+^, Ca^2+^, Glc, Fru, Mal) with concentrations 30 times that of AA.

AA, a strong antioxidant, is widely used in cosmetics, medicine, and the human diet. However, excessive intake of AA can also lead to many diseases, such as the common cold, scurvy, mental illness, and cancer [26]. AA can reduce oxTMB to TMB, accompanied by color fading in the solution. Therefore, based on the catalytic activity of Mn-CeO_2_, an efficient visual colorimetric sensing platform for detecting AA is established. As shown in Figure 13a, as the AA concentration increases, the reaction system’s absorbance gradually decreases at 652 nm. Figure 13b indicates that the concentration range of AA is 1.0~40.0 μM; there is a good linear relationship between the absorbance of the reaction system and the concentration of AA. The linear equation is A = −0.01121C_AA_ + 0.81267 (R^2^ = 0.992), the limit of detection (LOD) for AA was calculated to be 0.98 μM at an S/N (signal/noise) ratio of 3. The comparison of AA and LOD ranges detected by nano-enzymes of different materials is shown in Table 6.

In addition to sensitivity, selectivity and anti-interference are also important criteria to measure whether the sensor system is excellent [27]. We used Leu, Gly, Thr, L-Cys, Trp, Na^+^, K^+^, Mg^2+^, Ca^2+^, Glc, Fru, and Mal, which may exist in organisms, to further study the selectivity and anti-interference of the method. As shown in Figure 13c, the concentration of ascorbic acid (AA) in the selectivity experiment was set to 10 μM, and the concentrations of these interfering substances were approximately 30 times that of AA (300 μM). No significant change in the absorbance of the reaction system was observed, which demonstrates that the sensing method exhibits excellent selectivity and interference resistance for AA detection. In order to verify the accuracy of testing AA content in real samples under complex environment, we conducted recovery and precision tests of adding AA solution of different standard concentrations to vitamin C samples, and further evaluated the accuracy of this colorimetric detection method. As shown in Table 7, the recoveries ranged from 98.7 to 104.2% and the RSD was less than 3%. This demonstrates that the method can be used for the accurate determination of AA content in actual samples.

**Table 6:** The linear range and LOD of AA were measured by nano-enzymes of different materials.

Material	Linear Range (μM)	LOD (μM)	Ref.
Cu-Ag/rGo	5~30	3.6	[28]
M-CQDs N-	10~75	3.26	[29]
CeO_2_@SiO_2_	0.5–4000	70.18	[30]
Mn-CeO_2_	1~40	0.98	This work

**Table 7:** Detection of AA in vitamin C samples.

Enzyme Mimics	Samples	Added AA (μM)	Found AA (μM)	Recover (%)	RSD (%)
12 Mn-CeO_2_	Vitamin C	10	10.42	104.2	2.63
		20	20.34	101.7	1.94
		30	29.62	98.7	1.62

The developed colorimetric method demonstrates significant advantages for real sample analysis. For H_2_O_2_ detection in milk, the present method achieves excellent recovery rates (98.4–102.8%) and precision (RSD < 3%), comparable to or better than recently reported nanozyme sensors [16,17,18,19]. For AA detection in vitamin C tablets, Mn-CeO_2_ provides high sensitivity (LOD: 0.98 μM), excellent selectivity against 30-fold interferents (Figure 13c), and simplified operation compared to complex instrumental methods [28,29,30]. These results demonstrate that Mn-CeO_2_ outperforms recently reported nanozyme sensors in terms of sensitivity, selectivity, and practical applicability [10,20,21,22,23,24,25,26,27,28,29,30].

### 3.5. Study on the Colorimetric Sensing Mechanism of H_2_O_2_ and AA by Mn-CeO_2_

Based on the above experiments, the reasons for the excellent peroxidase-like activity of Mn-CeO_2_ can be inferred as follows: firstly, CeO_2_ with nanoflower morphology has good dispersibility and numerous active sites. Secondly, under the same conditions, the electron-hole pair provided by Mn itself co-forms a REDOX system with Ce^3+^/Ce^4+^, which is conducive to improving the electron migration rate. Mn-CeO_2_ showed better catalytic performance than CeO_2_ alone. Thirdly, due to the competition of multiple metal cations (Ce^3+^/Ce^4+^, Mn^2+^/Mn^3+^/Mn^4+^) for ligands, the coordination type of the central metal ion of CeO_2_ is changed, the active site and specific surface area are increased, and Mn-CeO_2_ enhances the interaction between substrate TMB and H_2_O_2_. Furthermore, the peroxide-like activity of CeO_2_ was increased.

**Figure 14:**
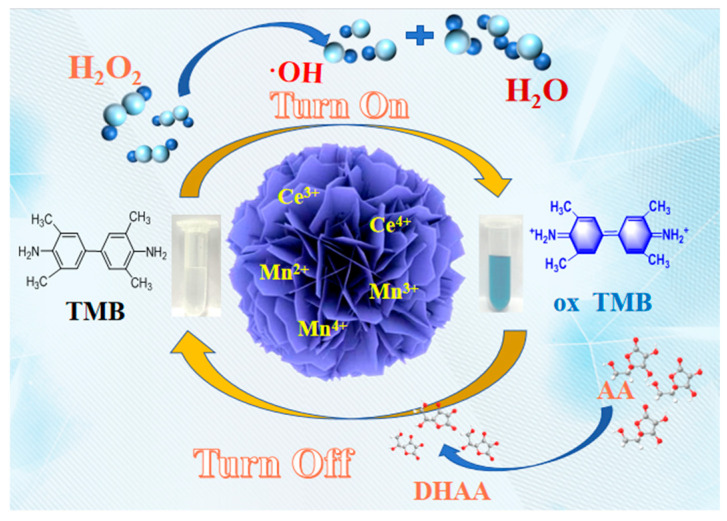
Mechanism of H_2_O_2_ and AA detection by Mn-CeO_2._

In addition, as shown in Figure 14, to clarify the correlation between the Ce^3+^/Ce^4+^ and Mn^2+^/Mn^3+^/Mn^4+^ redox cycles and the specific steps of H_2_O_2_ decomposition, the peroxidase-like catalytic mechanism of 12% Mn-CeO_2_ was systematically elaborated based on the coexistence of Ce^3+^/Ce^4+^ and Mn^2+^/Mn^3+^/Mn^4+^ confirmed by XPS, as well as the ·OH generation verified by ESR. Specifically, the catalytic process involves three synergistic reaction steps: first, H_2_O_2_ molecules are initially adsorbed on the Mn^2+^ active sites of Mn-CeO_2_, where Mn^2+^ acts as an electron donor and is oxidized to Mn^3+^, while H_2_O_2_ undergoes heterolysis upon accepting electrons to generate ·OH and OH^−^, which is consistent with the classic pathway of Mn^2+^ oxidation to Mn^3+^ by H_2_O_2_ in manganese peroxidase (MnP) and provides the electron transfer basis for initial ·OH formation. Second, the significantly increased Ce^3+^ content on Mn-CeO_2_ surface exhibits strong reducibility, which reduces the generated Mn^3+^ back to Mn^2+^ while Ce^3+^ is oxidized toCe^4+^, completing the regeneration of Mn^2+^ active sites; the regenerated Mn^2+^ can continuously react with H_2_O_2_ to produce ·OH, and meanwhile, Ce^4+^ as an oxidant can directly react with H_2_O_2_ to form Ce^3+^ and ·OH, constructing a synergistic electron transfer chain of “Ce^3+^/Ce^4+^-Mn^2+^/Mn^3+^”. Third, a portion of Mn^3+^ in the system can be further oxidized to Mn^4+^ by H_2_O_2_, and Ce^3+^ can also reduce Mn^4+^ to Mn^2+^ or Mn^3+^, forming a closed-loop cycle of Mn valence states and avoiding the deactivation of active sites caused by the accumulation of high-valent Mn ions. Collectively, the synergistic redox cycles of Ce^3+^/Ce^4+^ and Mn^2+^/Mn^3+^/Mn^4+^ not only reduce the activation energy barrier of H_2_O_2_ decomposition through continuous electron transfer but also achieve efficient regeneration of active sites, ensuring the sustained generation of ·OH. This mechanism forms a complete logical closed-loop with the ESR detection results and the enhanced catalytic activity of Mn-CeO_2_, providing a clear and scientific explanation for the peroxidase-like behavior of Mn-CeO_2_ [31,32].

## 4. Conclusions

In this work, flower-like M-CeO_2_ nanomaterials were synthesized and Mn-CeO_2_ with the optimal peroxidase-like activity was screened out from a series of metal-doped CeO_2_ composites. The effects of different Mn doping ratios on the peroxidase-like activity of the Mn-CeO_2_ solid solutions were systematically investigated. The 12% Mn-CeO_2_ exhibited the highest catalytic activity, which originated from the synergistic electron-hole pairs between Mn^2+^ and the Ce^3+^/Ce^4+^ redox couple, facilitating electron transfer and following the ·OH radical mechanism. The steady-state kinetics obeyed the Michaelis-Menten equation. Based on the excellent peroxidase-mimicking properties of Mn-CeO_2_, a highly sensitive colorimetric method was established for the detection of H_2_O_2_ and AA. This method was successfully applied to the determination of H_2_O_2_ in skim milk and AA in vitamin C tablets with high accuracy and precision, thus providing a promising strategy for food safety analysis and related analytical chemistry applications.

## Figures and Tables

**Table 1:** Lattice parameters of pure CeO_2_ and Mn-CeO_2_ with different doping ratios.

Sample	2θ (111) (°)	Lattice Parameter a (Å)
Pure CeO_2_	28.60	5.411
3% Mn-CeO_2_	28.62	5.409
6% Mn-CeO_2_	28.64	5.407
9% Mn-CeO_2_	28.67	5.404
12% Mn-CeO_2_	28.70	5.402
15% Mn-CeO_2_	28.72	5.399

**Table 2:** Specific surface area, pore volume, and pore size of CeO_2_ and 12Mn-CeO_2_.

Material	S_BET_ (m^2^·g^−1^)	V_P_ (cm^3^·g^−1^)	D_p_ (nm)
CeO_2_	71.97	0.059	3.412
12Mn-CeO_2_	91.22	0.056	3.411

**Table 3:** K_m_ and V_max_ comparison of nanozymes.

Catalyst	H_2_O_2_		TMB		Ref.
	K_m_ (mM)	V_max_ (10^−8^ M·S^−1^)	K_m_ (mM)	V_max_ (10^−8^ M·S^−1^)	
HRP	3.7	8.71	0.434	10	[17]
Fe_3_O_4_ MNPs	154	9.87	0.098	3.44	[18]
6Fe/CeO_2_	47.6	8.6	0.176	16.6	[19]
12 Mn-CeO_2_	2.588	16.89	0.156	20.50	This work

**Table 4:** The linear range and LOD of H_2_O_2_ were measured by nano-enzymes of different materials.

Material	Linear Range (μM)	LOD (μM)	Ref.
Fe-Ag_2_S	10~150	7.82	[20]
CuZnFeS	10~55	3	[21]
Fe_3_O_4_/MnO_2_	0.5~77	6	[22]
PDI-CeO_2_	20~80	2.59	[23]
His-AuNCs	5~135	3.6	[24]
Ni-MOF	0.1~20,000	4	[25]
Mn-CeO_2_	2~120	1.7	This work

**Table 5:** The content of H_2_O_2_ in skim milk was determined.

Enzyme Mimics	Samples	Added H_2_O_2_ (μM)	Found H_2_O_2_ (μM)	Recover (%)	RSD (% n = 3)
12 Mn-CeO_2_	Skim milk	50	50.3 8.71 0.434	100.6	2.25
		100	98.4 0.098	98.4	1.96
		150	154.2	102.8	1.78

## Data Availability

The original contributions presented in this study are included in the article. Further inquiries can be directed to the corresponding author.
